# An Efficient Strength Evaluation Method Based on Shell-Fastener Model for Large Hybrid Joint Structures of C/SiC Composites

**DOI:** 10.3390/ma17236008

**Published:** 2024-12-08

**Authors:** Maoqing Fu, Jiapeng Chen, Ben Wang, Biao Wang

**Affiliations:** 1 School of Materials Science and Engineering, Dongguan University of Technology, Dongguan 523808, China; fmq@dgut.edu.cn (M.F.); wangben0909@163.com (B.W.); 2 Guangdong Provincial Key Laboratory of Extreme Conditions, Dongguan 523803, China; 3 School of Physics and Sino-French Institute of Nuclear Engineering and Technology, Sun Yat-sen University, Guangzhou 510275, China

**Keywords:** C/SiC, hybrid joint structure, fastener, strength envelop method

## Abstract

C/SiC composites are widely used in aerospace thermal structures. Due to the high manufacturing complexity and cost of C/SiC composites, numerous hybrid joints are required to replace large and complex components. The intricate contact behavior within these hybrid joints reduces the computational efficiency of damage analysis methods based on solid models, limiting their effectiveness in large-scale structural design. According to the structure characteristic, a fractal contact stiffness model considering bonded behaviors is established in this paper. By introducing this model, it is proved that the bonded layer can affect the interface strength between plates but not the bearing strength of the specimen for the bolt/bonded hybrid joint structure. Furthermore, by introducing the strength envelope method, this paper overcomes the problem wherein the shell-fastener model cannot accurately describe the complex stress field. Validation through experimental comparison confirms that this approach can accurately predict both the failure mode and strength of multi-row hybrid joint structures in C/SiC composites at a detailed level with an error of 5.4%, including the shear failure of bolts. This method offers a robust foundation for the design of large-scale C/SiC composite structures.

## 1. Introduction

In the aerospace field, C/SiC composites, prepared by processes such as polymer impregnation pyrolysis (PIP) [1,2,3], chemical vapor infiltration (CVI) [4,5,6] and other processes [7,8] are considered ideal thermal protection materials and are widely applied in various thermal structures [9,10,11]. However, due to the high complexity and cost of manufacturing C/SiC, many mechanical joints are used to replace large, complex components. To ensure spacecraft reusability, bolted joint structures serve as the main type of mechanical connection. For consistent thermal expansion, all joint components are made from C/SiC and are produced with gas-phase CVI-C/SiC connection technology. After mechanical assembly, a secondary deposition process forms SiC bonded layers on each contact interface. This approach gives C/SiC joint structures both material uniformity and hybrid joint characteristics.

**Figure 1:**
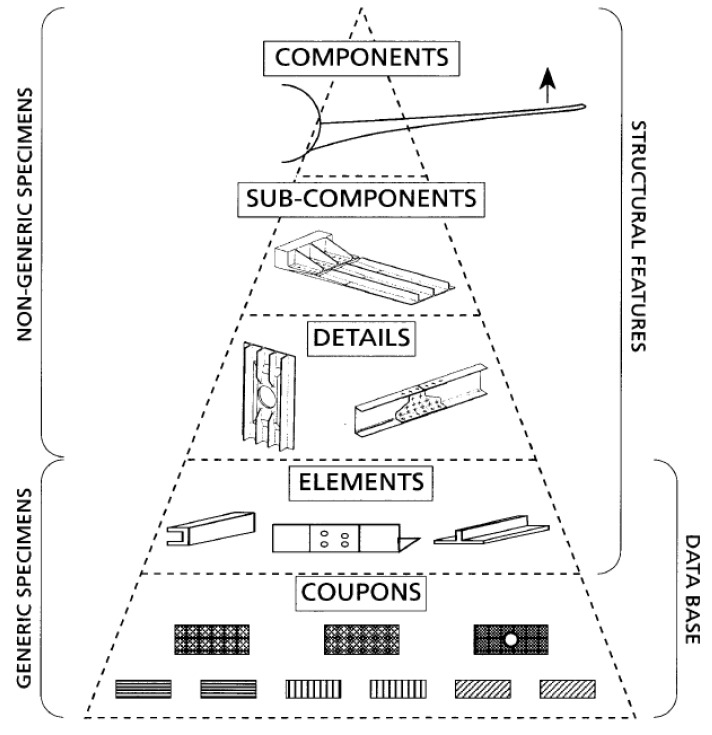
The “pyramid” of tests in the “building-block” approach [13].

As the weak point in the overall structure, C/SiC hybrid joint structures play a crucial role in maintaining structural integrity. Studying these joints is essential for the design and application of such structures. In response, the European Union launched the BOJACS (Bolted Joints in Composite Aircraft Structures) program [12], aiming to guide the design of large composite joint structures. *The Composite Materials Handbook* [13] introduces a “pyramid” of tests in a “building-block” approach for ceramic matrix composite joints, as shown in Figure 1. Ceramic matrix joint structures are divided into five levels based on structural complexity with design advancing step by step. This approach relies on foundational research at the coupon and element levels, where considerable progress has been made. Many studies have focused on experimental and numerical simulations of mechanical joints for different material systems [14,15,16,17]. However, research at levels above the detail level has reached a bottleneck, which is mainly due to high experimental costs and limited computational efficiency. To further study detail-level joint structures using numerical simulations, developing equivalent modeling methods is crucial to improve computational efficiency while maintaining analysis accuracy and validity.

The key to achieving model equivalence is dimensionality reduction. Early approaches reduced only the fastener’s dimension to a 1D beam or truss element to capture the complex stress state at the hole edge and constrain relative movement between plates [18]. However, without reducing the plate’s dimension, computational efficiency remained low when analyzing components above the detail level. McCarthy et al. [19] introduced a user-defined element model that simplified the joint structure into a shell-fastener model, combining a 2D shell with a 1D connection element using multipoint constraint (MPC) technology. This model significantly improved computational efficiency and, with continuous optimizations by various researchers [20,21,22,23], can now capture different forms of relative deformation between plates and accurately calculate bolt loads. For C/SiC hybrid joint structures, some scholars have carried out systematic experimental research, studied the damage failure mechanism, and explored the modeling method [24,25]. However, due to the limitations of the model and the computational conditions, they still could not introduce the contact behavior of the bonded layer and the high roughness surface into the model. Fractal contact models, widely used to address complex surface interactions, can describe the actual contact area and stiffness without requiring intricate surface modeling [26,27,28]. On this basis, we developed a modified equivalent stiffness model that considers the distribution of the SiC bonded layer and its impact on structural strength [29,30]. However, this model does not take into account the mixed contact behavior of bonded and fractal contact, and it omits detailed hole and fastener features, limiting its use in progressive damage analysis [31,32,33], which would also demand significantly more computation—opposing the goal of efficiency in equivalent models.

The strength envelope method, introduced by Hart-Smith [34], predicts the strength of bolted joint structures by focusing on the distribution relationship between the bearing load and the bypass load. In this paper, we combine the shell-fastener model with the fractal contact stiffness model for C/SiC composite hybrid joints and incorporate the strength envelope method to create a comprehensive approach for evaluating the static strength of large components beyond the detail level. Experimental results confirm the validity of this method, offering a reliable basis for designing large-scale C/SiC composite structures.

## 2. Experiments

### 2.1. Specimen and Tensile Test of C/SiC Composite Multi-Row Hybrid Joint Structures

**Figure 2:**
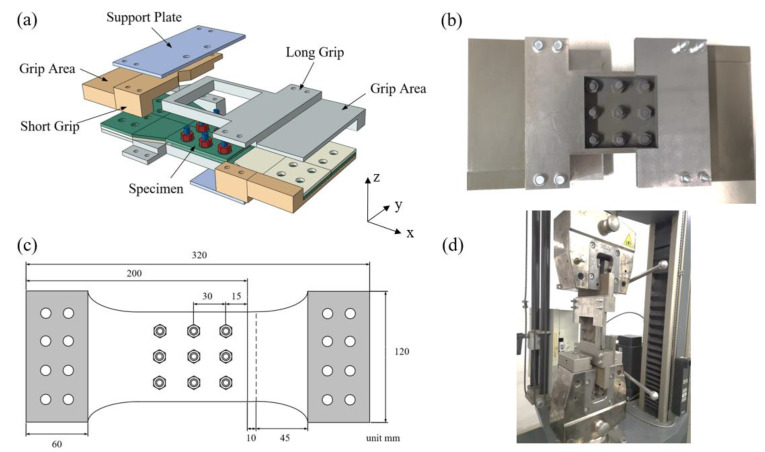
Tensile test of C/SiC composite multi-row hybrid joint structures: (**a**) anti-deflection fixture; (**b**) specimen assembled anti-deflection fixture; (**c**) specimen of C/SiC composite three-row, single-lap, three-bolt joint; (**d**) clamping method.

Due to the high cost of C/SiC composite hybrid joint structures, we fabricated two specimens for tensile test. It is used as the data basis for numerical simulation analysis. The material properties of the C/SiC composites are listed in Table 1, and the configuration and dimensions of the specimens are illustrated in the Figure 2c. The pitch of the bolts in the x and y directions is 30 mm, and the thickness of the joint plate is 4 mm. The preload torque is 2 Nm. To prevent bolt shear failure, 8 mm diameter bolts were selected [35]. Given that the secondary bending in single-lap joints cannot be ignored, an anti-deflection fixture was designed and fabricated in accordance with ASTM D5961 [36]. As shown in Figure 2a, this fixture consists of three components: a long fixture, a short fixture, and a support plate. The separate design of the long and short fixtures allows for tensile deformation of the specimen, while the slotted design of the long fixture facilitates displacement measurement and accommodates the lateral displacement caused by the bolts. The support plate, in clamping the fixtures, also restricts them from bending along with the specimen’s secondary bending, preventing vertical displacement and serving an anti-buckling function. Figure 2b shows a specimen assembled in the anti-deflection fixture. According to ASTM D5961 and GJB 6475-2008 [37] standards, tensile failure tests were conducted using a Zwick Z100 universal testing machine (Team Testing Equipment, Guangzhou, China), with a measurement range of 0 to 100 kN, as shown in Figure 2d. Displacement was recorded using an extensometer. The tests were conducted at room temperature in displacement control mode at a speed of 0.2 mm/min. The tests were terminated once the specimens were fully unloaded.

**Figure 3:**
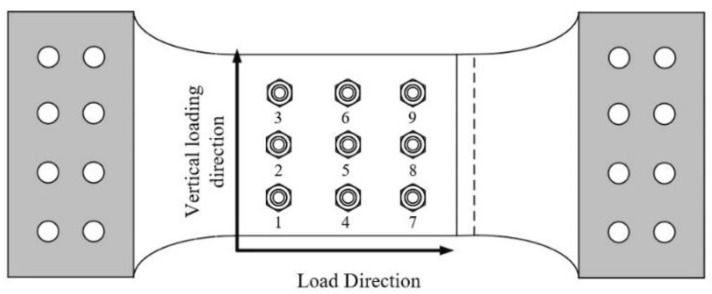
Numbering rule of bolts.

Figure 3 illustrates the bolt numbering scheme. In the loading direction, the bolts are numbered sequentially from farthest to nearest as the first, second, and third rows. Perpendicular to the loading direction, the numbering increases from bottom to top.

### 2.2. Results of Tension Tests

#### 2.2.1. Results of Specimen 1

**Figure 4:**
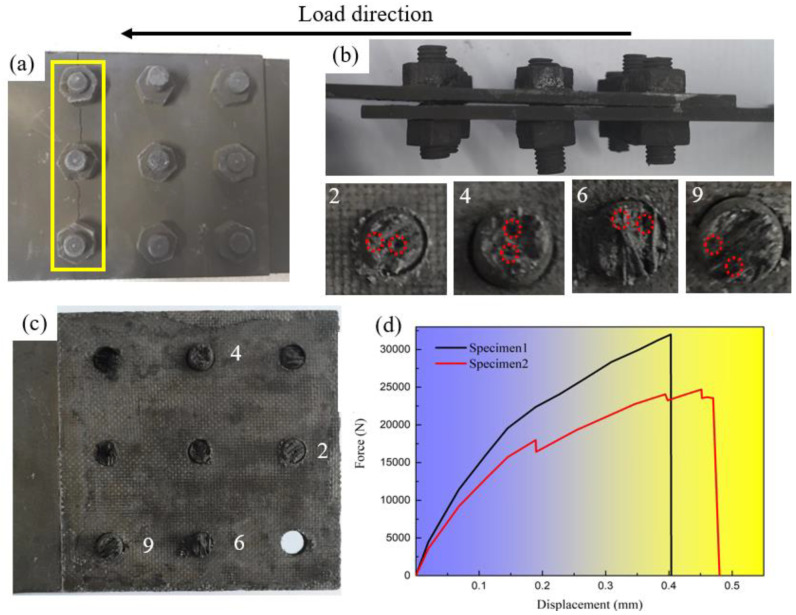
Results of tensile tests: (**a**) tensile failure mode of specimen No. 1; (**b**) tensile failure mode of specimen No. 2; (**c**) surface morphology of lap joint area; (**d**) load–displacement curves.

Before complete unloading, a snapping sound was heard, and shortly after, Specimen 1 failed, marking the end of the test. As shown in Figure 4a, the failure mode of Specimen 1 was consistent with tensile failure at the joint plate near the first-row bolts. The load–displacement curves for both specimens are presented in Figure 4d. The tensile failure load for Specimen 1 was recorded as 31,997.3 N.

#### 2.2.2. Results of Specimen 2

Before failure, three distinct snapping sounds were heard during the test. As shown in Figure 4b,c, three unloadings corresponded to the shear failure of the bolts in each of the three rows with each snapping sound indicating the shearing of an entire row of bolts. Notably, the joint plates remained undamaged. The first unloading occurred at a load of 17,977.2 N, corresponding to the shear failure of the first row of bolts. The second unloading took place at 23,239.1 N, corresponding to the shear failure of the second row. Finally, after the third row of bolts sheared, the specimen reached a failure load of 24,700.5 N. As the number of sheared bolts increased, the load-bearing capacity of the specimen progressively decreased, leading to an accelerated rate of failure.

## 3. Simulation

### 3.1. Influence of the Bonded Layer on the Bearing Strength of the Bolt/Bonded Hybrid Joint Structure

**Figure 5:**
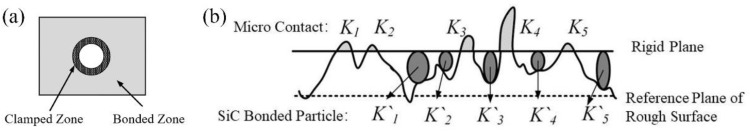
Equivalent contact stiffness: (**a**) interface of bolted hybrid joints; (**b**) parallel stiffness model of clamping area.

In bolted joint structures, the actual clamped zone (nominal contact area) between the joint plates forms a circular region at the interface, as shown in Figure 5a, while the remaining areas are not in contact. During the second deposition of the specimen, a bonded layer develops in the non-contact gray region, as illustrated in Figure 5a. Depending on the extent of the second deposition, the black clamped zone may also become infiltrated by SiC particles, forming a mixed zone. Additionally, the relative density distribution of the SiC bonded layer along the joint plate contour decreases from the outer edge toward the center. As a result, the porosity of the adhesive layer in the black mixed zone is likely to be higher than in the gray region.

Based on the micro-contact between asperities, SiC particles adhered to the pores that were not in contact, resulting in a parallel combination of asperity contact stiffness and the bonded stiffness of the SiC particles at the interface of the mixed zone, as illustrated in Figure 5b. The equivalent contact stiffness is expressed in Equation (1):

(1) $$\begin{array}{l} K_{h}=\left(K_{1}+K_{2}+K_{3}+K_{4}+K_{5}\right)+\left({K^{\prime }}_{1}+{K^{\prime }}_{2}+{K^{\prime }}_{3}+{K^{\prime }}_{4}+{K^{\prime }}_{5}\right)\\ K_{h}=\sum_{i=1}^{n}K_{i}+\sum_{j=1}^{m}K_{j} \end{array}$$

where $\sum_{i=1}^{n}K_{i}$ is fractal contact stiffness [29]; and $\sum_{j=1}^{m}K_{j}$ is the total bonded stiffness whose value is related to porosity [30].

**Figure 6:**
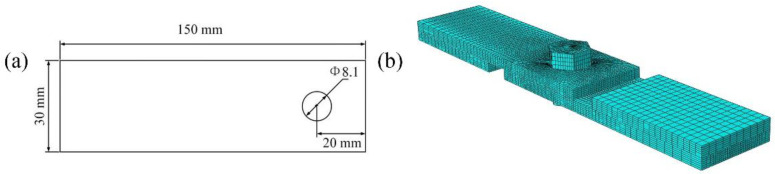
C/SiC bolted-bonded hybrid single-lap joint structure: (**a**) size of C/SiC composite joint plate; (**b**) finite element model.

To examine the effect of the bonded layer on the bearing strength of the bolt/bonded hybrid joint structures, the bearing failure process of a single-bolt, single-lap hybrid joint structure, as shown in Figure 6, was simulated. We used the commercial software ABAQUS version 6.14 for numerical simulation. An 8-node hexahedral solid element (C3D8) was used, and the five parts had a total of 50,748 elements. The clamping region of one end was constrained by fixed support, and the clamping end of the other segment was constrained by all degrees of freedom except the degree of freedom in the load direction. A displacement load of 2 mm was applied in the load direction. The fractal contact model considering the bonded layer (nonlinear contact stiffness model) is input according to Equation (1). The friction coefficient of the C/SiC composite is 0.243, and before we simulate the tensile test, we firstly simulate the local deformation and stress state of the bolt after tightening. To account for the impact of porosity on the strength of the adhesive layer, two representative porosity levels, 40% and 55%, were selected as input for the material properties [30].

**Figure 7:**
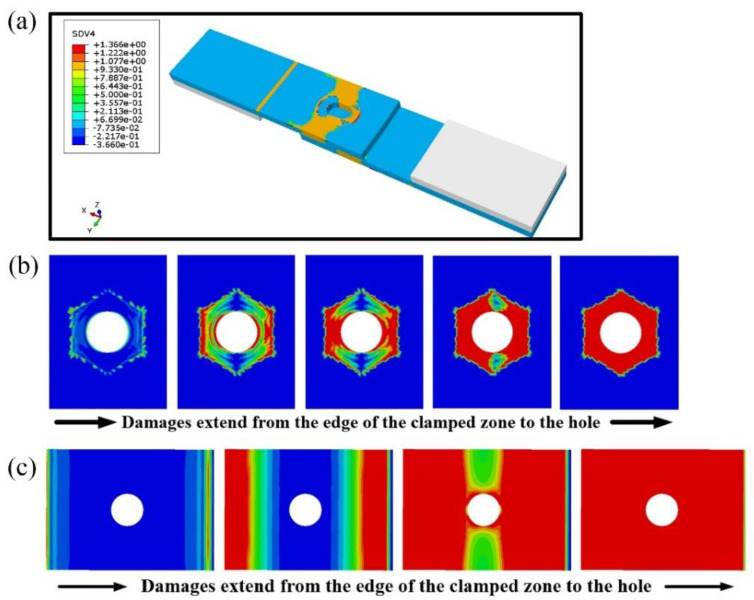
Simulation results: (**a**) bearing damage distribution; (**b**) damage expansion of bonded layer between nut and plate; (**c**) damage expansion of bonded layer between plate and plate.

The calculated bearing damage simulation is shown in Figure 7a. Due to the load applied by the bolt, the loaded hole elongates significantly in the direction of compression, creating a notch and resulting in evident bearing failure. The local area around the hole and the clamped end also exhibit varying degrees of damage. Figure 7b shows the simulated results of the damage propagation process in the bonded layer between the nut and the plate. Since the selected bolt is hexagonal, the contact interface forms a regular hexagon. As the tensile load increases, debonding begins at the edge of the hole in the load direction. This debonding occurs simultaneously in the direction perpendicular to the load. The debonded area expands from the load direction toward the perpendicular direction, progressively spreading along the hole’s edge until complete debonding occurs, leading to a transitional phase. Figure 7c illustrates the simulated damage propagation process in the bonded layer between the plates. Similar to the debonding process with fully bonded pins [30], the debonded area first spreads from the edge in the load direction toward the hole’s edge. Once debonding is complete at the edge, it extends into the region perpendicular to the load, eventually leading to complete debonding and entering a transitional phase.

**Figure 8:**
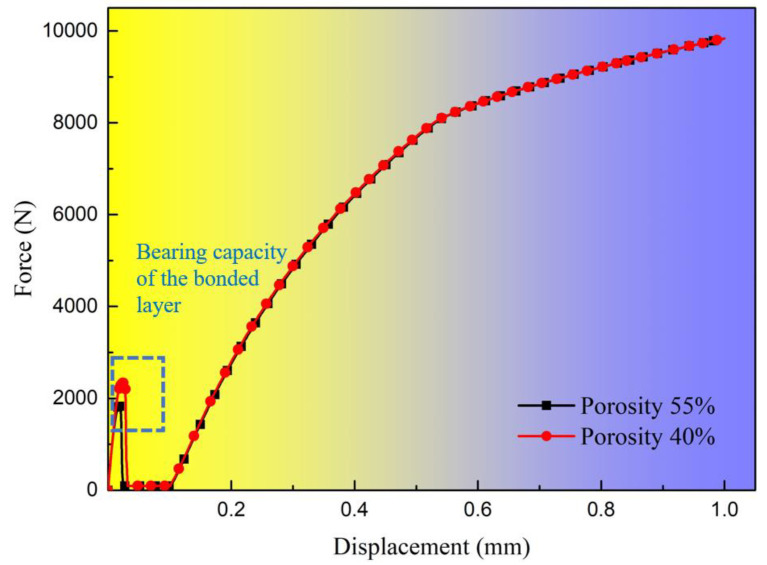
Tensile load–displacement curves of specimens with different porosity bonded layers.

Figure 8 illustrates the tensile load–displacement curves for simulations with porosities of 55% and 40%. It is evident that due to the presence of a clearance fit, the tensile load does not induce mutual compression between the bolt and the hole before the adhesive layer fails. Consequently, the bonded layer does not influence the bearing strength of the single-bolt, single-lap bolt/bonded hybrid joint structures under clearance fit conditions. However, bonded layers with different porosities will affect the bearing capacity of the bonded layer. Once complete debonding occurs, the bolt transitions into a transitional phase. Thus, the material properties of the bonded layer primarily influence the shear stiffness in the quasi-linear segment of this type of hybrid joint structure with bonded layers exhibiting lower porosity enhancing the shear stiffness within a specific range of the quasi-linear phase.

### 3.2. Simulation of Tensile Test by Shell-Fastener Model

**Figure 9:**
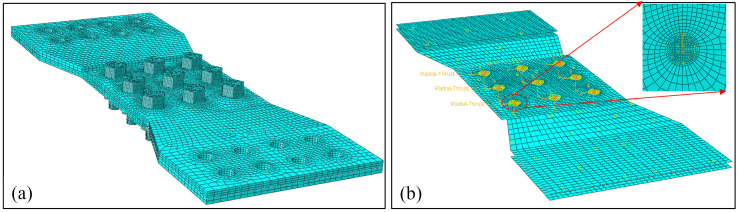
Finite element model of C/SiC composite three-row, three-pin single-lap bolted joint specimen: (**a**) solid model; (**b**) shell-fastener model.

Based on the analysis in Section 3.1 and the experimental results presented in Figure 4d, the unloading section in Figure 8 was not found in the experimental curves of the two specimens. Therefore, both specimens can be considered to ignore the effect of the bonded layer. This conclusion is further supported by Figure 4c, indicating that the bonded layer can be neglected in the calculations. Figure 9 illustrates the finite element model of a C/SiC composite three-row, three-pin single-lap bolted joint specimen. In Figure 9a, the three-dimensional solid model is converted into the shell-fastener model shown in Figure 9b using a rapid equivalent modeling method [38]. The bolts are represented by the fastener model, which connects the two joint plates through connector elements, with multi-point coupling in the relevant areas representing the interaction between the bolts and the plates. In order to reflect the deformation of the hole edge, 10 parts are divided every quarter circle, and the minimum element length is 0.628 mm. The plate model is discretized using four-node reduced integration three-dimensional shell elements (S4R), which has a total of 15,412 elements. Due to the implementation of anti-shear fixtures, the entire specimen does not experience out-of-plane deformation; constraints are applied to prevent out-of-plane translation and rotation, while complete constraints are placed on the clamped area, allowing only translational freedom in the tensile direction at the loading end.

Due to the complexity of the actual stress field in C/SiC composite multi-row hybrid joint structures under load, the shell-fastener model is insufficiently accurate for describing the stress field. The precision of the stress field is critical for progressive damage analysis. Therefore, it is necessary to develop a strength prediction method suitable for equivalent models. The strength envelope method can accurately predict the failure mode and strength of the structure by simply obtaining information on bolt load distribution. In the following section, this paper will utilize the strength envelope method [34] to evaluate the static strength of C/SiC composite multi-row hybrid joint structures. Since the strength envelope method requires only the load ratio information for critical holes to predict the failure load and mode, the simulation can focus on obtaining pin load data to calculate the load ratios based on external loads. Consequently, a smaller load value can be specified for the calculations with a tensile force of 18,000 N designated for this case. This specimen can be regarded as three single-row, three-bolt specimens connected in parallel. The design of the spacing (with row spacing greater than or equal to the spacing of the bolts) ensures that the mutual influence between each row of bolt connections is negligible. Therefore, as indicated in Table 2, the positions of the connecting plates corresponding to bolts No. 1, No. 2, and No. 3 are all critical locations, and the stress ratio by Equation (3) for these three bolts is 1.375. Consequently, the failure load of a representative bolt can be calculated using the strength envelope method, and the resulting value will be recorded as the final failure load for Specimen No. 1.

## 4. Discussion

### 4.1. Static Strength Analysis Based on Strength Envelope Method

**Figure 10:**
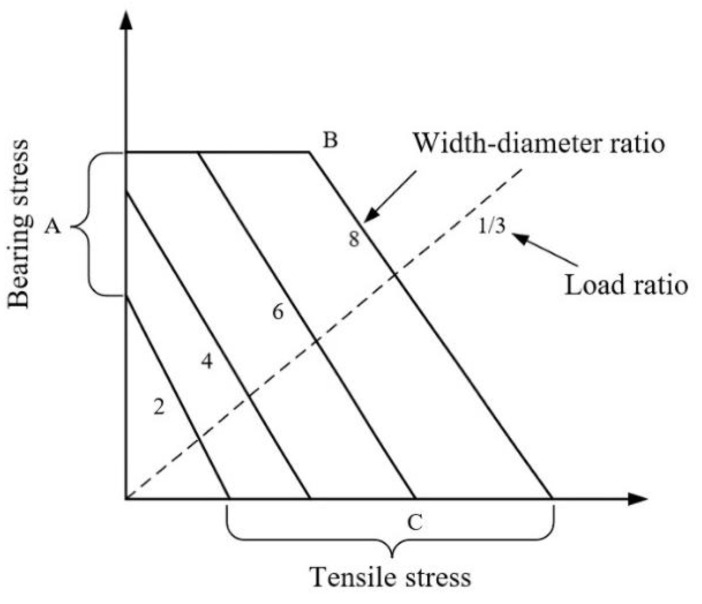
Strength envelope of typical mechanical joint structures.

**Figure 11:**
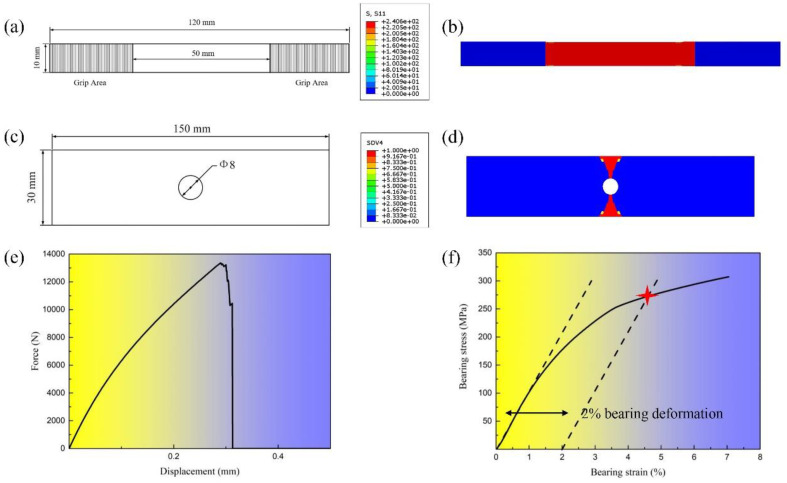
The various strengths required to draw the strength envelope: (**a**) the size of C/SiC composite plate; (**b**) tensile strength of C/SiC composite plate; (**c**) the size of plate with opening hole; (**d**) tensile failure process simulation of plate with opening hole; (**e**) tensile load–displacement curve of plate with opening hole; (**f**) bearing strength of specimen (red star which represents the intersection of the 2% bearing deformation and the curve).

**Figure 12:**
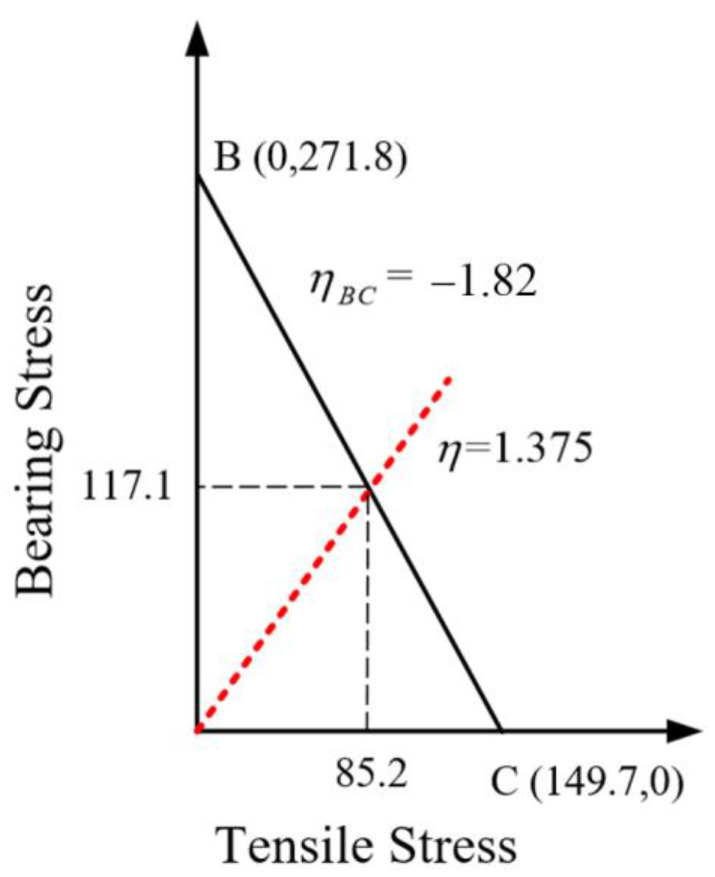
Tensile strength envelope of C/SiC composite three-row, three-pin single-lap bolted joint specimen.

Figure 10 illustrates the strength envelopes for typical joint structures, including those with bolt diameter-to-width ratios of 2, 4, 6, and 8. The vertical axis represents the bearing stress, with point A indicating the intercept of each envelope on this axis, reflecting the bearing strength at the loading hole. The horizontal axis represents the tensile stress (or bypass stress), where point C denotes the intercept of each envelope on this axis, representing the tensile strength of the plate with holes. The slope of the solid line BC represents the ratio of the two stress concentration reduction factors $\eta_{BC}$, which are expressed as follows:

(2) $$\{\begin{array}{l} \eta_{BC}=-\frac{K_{tc}}{K_{bc}^{j}}\\ K_{tc}=\frac{\sigma_{b}}{P_{ult}/\left(W-D\right)t}\\ K_{bc}^{j}=\frac{\sigma_{b}}{P_{bru}^{j}/Dt} \end{array}$$

where $K_{tc}$ is the concentration reduction coefficient of tensile stress in composite plates with holes, $\sigma_{b}$ is the tensile strength of the plates, $P_{ult}$ is the tensile failure load of plates with holes, $P_{bru}^{j}$ is the bearing failure load of plates with loading holes, $K_{bc}^{j}$ is the concentration reduction coefficient of bearing stress in composite plates with loading holes. *W*, *D* and *t* represent the width, bolt diameter, and thickness, respectively. The slope of the dashed line represents the stress ratio of each bolt, which is expressed as

(3) $$\eta =\frac{\sigma_{br}^{j}}{\sigma_{net}}=\frac{P_{br}^{j}/D}{P_{by}/\left(W-D\right)}=\frac{\left(W-D\right)}{D}R$$

where $\sigma_{br}^{j}$ is the bearing stress of the loading hole, $\sigma_{net}$ is the tensile stress of the net section, $P_{br}^{j}$ is the bolt load, $P_{by}$ is the bypass load, and *R* is the load ratio. Point B represents the condition where tensile failure and bearing failure occur simultaneously. When the load ratio is less than this point, tensile failure occurs, while a load ratio greater than this point leads to bearing failure. It is evident that when the width-to-diameter ratio is less than 4, the strength envelope method indicates that tensile failure will not occur in non-single-bolt joint structures. The intersection of the dashed line with the strength envelope provides the bearing and bypass loads corresponding to the bolt’s failure. For single-row joint structures, the sum of the bearing and bypass loads constitutes the failure load. Therefore, in order to draw the strength envelope of the structure, the corresponding strength values need to be known first, and each strength value is determined by the finite element method; the corresponding results are shown in Figure 11, where $\sigma_{br}^{j}$ (point B) is 271.8 MPa, $\sigma_{net}$ (point C) is 149.7 MPa, and $\eta_{BC}$ is −1.82. Then, the strength envelope of the C/SiC composite multi-row hybrid joint structure is drawn as shown in Figure 12.

### 4.2. Failure Analysis Based on Shell-Fastener Model

This specimen can be considered as three single-row, three-bolt specimens connected in parallel. The design of the spacing (with row spacing greater than or equal to the spacing of the bolts) ensures that the mutual influence between each row of bolt connections is negligible. Therefore, as shown in Table 2, the positions of the joint plates corresponding to bolts No. 1, No. 2, and No. 3 are all critical locations, and the stress ratio for these three bolts is 1.375. Consequently, the failure load of a representative bolt can be calculated using the strength envelope method, and the resulting value will be recorded as the final failure load for the specimen.

As illustrated in Figure 12, the red dashed line represents the stress ratio line for bolt No. 1. The bearing stress and bypass stress corresponding to the point where it intersects the strength envelope are 85.2 MPa and 117.1 MPa, respectively. The calculated total load is 11,243.5 N, resulting in a final failure load of 33,730.5 N. Thus, the tensile failure load of the C/SiC composite multi-row hybrid joint specimen, determined using the strength envelope method, is 33,730.5 N, with an error of 5.4% compared to the test value for specimen No. 1. This indicates that the strength envelope method is suitable for analyzing the bolt joint structures of C/SiC composites. However, while the failure load and failure mode predicted by the strength envelope method align well with the test results for specimen No. 1, they fail to simulate the test phenomena observed in specimen No. 2 and cannot accurately predict the shear failure mode of the bolts.

As shown in Figure 4b,c, the failure mode of Specimen 2 was characterized by the shear failure of all bolts. The strength envelope method, however, is limited to predicting tensile and bearing failures of the joint plate and could not account for this shear failure mode. Based on findings from Ref. [33], the shear strength of C/SiC composite fasteners exceeds *S*_xy_: the in-plane shear strength of C/SiC composites, which is 114.5 MPa, is lower than their tensile strength of 238.9 MPa. According to the lower limit of 114.5 MPa, the maximum shear load for an 8 mm diameter bolt is calculated to be 5752.5 N, which is significantly higher than the 3555 N experienced by each bolt during tensile failure in specimen No. 1. This indicates that shear failure of the bolts would not occur as long as the bolt strength requirements are met. Therefore, it can be inferred that the actual strength of the first row of bolts must have been considerably less than 114.5 MPa due to manufacturing inconsistencies, leading to shear failure at a relatively low load. As fewer bolts carried the load, the load on the remaining bolts increased. Once this exceeded their ultimate load capacity, shear failure propagated until all bolts failed, resulting in the complete failure of specimen No. 2 and concluding the experiment. In addition, it can be clearly found from Figure 4c that many pores can be seen at the fracture of the bolt No. 2, 4, 6 and 9, which proves that there are obvious quality problems in this batch of bolts. This quality problem is often encountered by CVI processes due to insufficient deposition time.

Since only three breaking sounds were recorded during the test, and as observed in Figure 4d, the tensile load–displacement curve of specimen No. 2 exhibited only three unloading processes without any fluctuations during these unloading processes, the following assumptions are made:

(1) Regardless of which bolts in each row fail first, all bolts within the same row will experience shear failure simultaneously.

(2) Prior to the failure of any row of bolts, the load is distributed evenly across that row and the other rows of bolts. Based on these assumptions, the average shear strength of each row of bolts can be calculated as presented in Equation (4):

(4) $$\sigma_{shear-i}=\frac{P_{i}}{\frac{9}{i}A_{b}}=\frac{iP_{i}}{9A_{b}}$$

where *i* is the unloading times and number of bolt rows, $P_{i}$ is the maximum load at the *i*-th unloading, and $A_{b}$ is the bolt cross-sectional area. According to Equation (4), the average shear strength of the three rows of bolts is 39.8 MPa, 102.8 MPa and 163.88 MPa, respectively. The three shear strengths are defined as the maximum shear strength of the fastener model and embedded into the shell-fastener model [27]; when the load it is subjected to reaches shear strength, the fastener element fails. Since the macroscopic failure mode of bolts is directly determined by the bolt load and has nothing to do with the stress field of the hole edge, the shear failure of bolts can be simulated by defining the constitutive model of the fastener model.

**Figure 13:**
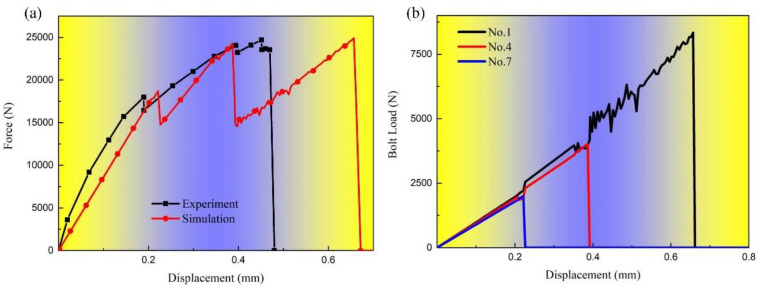
Simulation results of shell-fastener model: (**a**) comparing the tensile load–displacement curves obtained by tests and shell-fastener model of specimen No. 2; (**b**) bolt load history curve of specimen No. 2.

The comparison between the calculation results and the test results is illustrated in Figure 13a. While the maximum loads recorded during the three unloading events are generally consistent, the amounts of unloading vary significantly. This discrepancy arises because in the fastener model, once a bolt completely fails, it no longer contributes to any bearing mode. Consequently, the load is entirely redistributed to the remaining bolts, and the unloading amount corresponds to the maximum load carried by the damaged bolts prior to failure. Figure 13b presents the load history curves for bolts No. 1, 4, and 7 in each row, demonstrating the equal distribution of load among the bolts. It is important to note that even after shearing, the bolts remain within the bolt holes and do not detach from the specimen, thus maintaining a certain level of bearing capacity. As a result, the unloading amount of the tensile load is relatively small. This issue highlights a calculation error inherent to the shell-fastener model, which does not account for secondary bearing effects.

Furthermore, due to the increased unloading amount, the displacement required for the shell-fastener model to achieve the next maximum load, given the same shear stiffness, is larger than what was observed in the tests. Thus, the displacement calculation from the shell-fastener model is also greater than the experimental results. Nonetheless, the primary advantage of this model is its ability to accurately predict the corresponding strength of the specimen at the moment of shear failure, provided that the bolt shear strength is known beforehand. Consequently, the shell-fastener model can effectively predict various failure modes, including plate tensile failure, bearing failure, and bolt shear failure.

## 5. Conclusions

This paper establishes a fractal contact stiffness model to address the complex contact behaviors in hybrid joint structures. Using this model, a simulation of a single-bolt, single-lap hybrid joint shows that the bonded layer’s strength does not affect the bearing failure strength. Instead, it mainly influences the initial slope of the force–displacement curve and the adhesive force at the interfaces.

Based on the first finding and an analysis of experimental curves and failure interfaces, it is inferred that the bonded layer in both specimens can be omitted in modeling. Tensile tests showed distinct failure modes for each specimen: specimen No. 1 exhibited tensile failure of the joint plates near the first row of bolts, away from the load direction, while specimen No. 2 experienced shear failure in all nine bolts. The choice of an 8 mm bolt diameter was intended to prevent bolt shear failure; however, the observed test results indicate this was due to the bolts’ low shear strength.

The strength envelope method serves as the static strength evaluation approach for the shell-fastener model. Its validity is confirmed through comparisons with specimen No. 1. Additional comparisons with specimen No. 2 show that if the bolts’ actual shear strength is known beforehand, the specimen’s strength under bolt shear failure can be accurately predicted regardless of displacement results. This allows the shell-fastener model to account for a broader range of failure modes.

This paper introduces the strength envelope method to predict static strength for bolted joint structures without accounting for complex stress fields. By combining this method with the shell-fastener equivalent model, we offer a robust and efficient static strength prediction technique for structures beyond the detail level, providing critical support for designing C/SiC composite hybrid joint structures.

## Figures and Tables

**Table 1:** Material properties of C/SiC composite and SiC.

C/SiC	**Elastic Properties**								
	*E*_xx_(GPa)	*E*_yy_(GPa)	*E*_zz_(GPa)	*G*_xy_(GPa)	*G*_yz_(GPa)	*G*_xz_(GPa)	*v*_xy_	*v*_yz_	*v*_xz_
	120	120	60	44.4	24	24	0.25	0.35	0.35
	**Strength (MPa)**								
	*X*_T_	*Y*_T_	*X*_C_	*Y*_C_	*Z*_T_	*Z*_C_	*S*_xy_	*S*_yz_	*S*_xz_
	238.9	238.9	409.4	409.4	60.3	120.2	114.5	34.7	34.7
SiC	Elastic modulus	*E*_0_(GPa)	350	Poisson ratio	*v*	0.2	Strength (MPa)	*σ_s_*	100.52

**Table 2:** Calculation results of each bolt.

Bolt	No. 1	No. 4	No. 7	No. 2	No. 5	No. 8	No. 3	No. 6	No. 9
Bolt load (N)	2000	2000	2000	2000	2000	2000	2000	2000	2000
Bypass load (N)	4000	2000	0	4000	2000	0	4000	2000	0
Load ratio *R*	0.5	1	-	0.5	1	-	0.5	1	-
Stress ratio *η*	1.375	2.75	-	1.375	2.75	-	1.375	2.75	-

## Data Availability

The original contributions presented in the study are included in the article, further inquiries can be directed to the corresponding authors.
